# Antibiotic Stewardship Program in a General Hospital in Abu Dhabi, UAE: Preparedness for the COVID-19 Pandemic

**DOI:** 10.7759/cureus.67270

**Published:** 2024-08-20

**Authors:** Kanika Vats, Kuldeep Singh, Seema Oommen

**Affiliations:** 1 Department of Research and Development, Healthcare Technical and Compliance Directorate, Emirates Classification Society (TASNEEF), Abu Dhabi, ARE; 2 Department of Management, School of Commerce and Management, Om Sterling Global University, Hisar, IND; 3 Department of Laboratory, Burjeel Medical City Co-Lab, Abu Dhabi, ARE

**Keywords:** antibiotic utilization, clinical outcomes, intervention strategies, infection control, antibiotic stewardship, antibiotic resistance, prescribing practices

## Abstract

Introduction

The COVID-19 pandemic has highlighted the critical need for resilient healthcare systems capable of swift response and adaptation, particularly in light of the ongoing global threat of antibiotic resistance. Hospitals in Abu Dhabi, UAE, are not exempted and must establish robust antibiotic stewardship programs capable of navigating any pandemic, ensuring judicious antibiotic use while maintaining high standards of care and optimal patient outcomes. This study seeks to evaluate the maturity levels of antibiotic stewardship programs in a general hospital to assess preparedness for such health crises. By analyzing data from non-surgical hospitalized patients in a specific age bracket, the study examines prescribing practices, program efficacy, and the hospital's overall readiness to manage infectious disease outbreaks. The findings will guide efforts to strengthen antibiotic stewardship and improve pandemic readiness across healthcare settings.

Methods

The retrospective observational study focused on non-surgical hospitalized patients aged 25-40 from January to December 2019. Data were collected from electronic medical records between March 2023 and February 2024, using a predefined set of International Classification of Diseases, 10th Revision, Clinical Modification (ICD-10-CM) codes related to respiratory tract infections, urinary tract infections, ventilator-associated pneumonia, and nosocomial infections. The study evaluated clinicians' prescribing habits, antibiotic consumption, stewardship interventions, and the overall impact on the healthcare system to assess the implementation and maturity levels of the antibiotic stewardship program.

Results

A study of 240 cases involving 229 patients revealed significant findings in antibiotic use and resistance patterns based on predefined criteria. The average duration of antibiotic use per patient was 6.23 days. Duplicate anaerobic therapy was identified in 4.58% of cases. *Escherichia coli*,* Klebsiella pneumoniae*,* Enterobacter *spp., and *Proteus *spp. showed reduced susceptibility to multiple antibiotics. *Citrobacter *spp. were fully resistant to one antibiotic and had low susceptibility to another. *Haemophilus influenzae*,* Salmonella *spp.,* Staphylococcus *spp.,* Streptococcus *spp., and *Enterococcus* spp.* *displayed varying degrees of reduced susceptibility. Of the cases, 91.66% (n = 220) received antibiotics within 24 hours of admission, with 98.63% (n = 217) receiving empirical therapy. Inaccurate empirical decisions correlated with longer hospital stays (4.45 versus 3.36 days). Appropriate antibiotic stewardship was observed in only 2.35% of cases during stays exceeding three days and 16.47% at discharge.

Recommendation

A further longitudinal study is recommended to compare how these results contribute to our understanding of the impact of the COVID-19 pandemic on antibiotic stewardship practices, resistance trends, and clinicians' prescribing habits in non-surgical hospitals in Abu Dhabi.

Conclusion

The review highlighted key aspects of existing stewardship practices. While most patients received empirical therapy, issues such as duplicate anaerobic therapy and a concerning decline in antibiotic susceptibility were identified. Inaccurate empirical decisions were associated with longer hospital stays. The limited instances of appropriate stewardship conduct suggest a need for better adherence to antibiotic management practices and enhanced preparedness for future healthcare challenges.

## Introduction

Antimicrobial stewardship is pivotal in combating antimicrobial resistance (AMR), a rising public health challenge that jeopardizes the effectiveness of critical treatments. Within hospital environments, robust antimicrobial stewardship programs (ASPs) are essential for optimizing antibiotic use, enhancing patient outcomes, and lowering resistance rates [1-4].

Antibiotic resistance, the ability of pathogens to withstand antibiotics that would typically eradicate or restrain their growth, is influenced by various factors. These factors include the level of resistance exhibited by the bacterial strain and its capability to withstand antibiotics through resistance mechanisms [5,6]. Microbial strains may inherently possess resistance or acquire it through horizontal gene transfer mechanisms such as plasmids, transposons, genetic elements, and bacteriophages or through genetic alterations within the bacterial cell itself, leading to cross-resistance [7]. The rapid proliferation of resistant microorganisms can occur if resistance genes are situated on mobile genetic elements such as plasmids. Biochemical mechanisms underlie resistance by shielding the bacterial cell wall from antibiotic effects, involving target modification, enzymatic breakdown, and the modulation of uptake through efflux pump proteins. Consequently, first-generation antibiotics are encountering resistance across various clinical contexts due to these natural processes [8].

The initial cases of antibiotic resistance were observed shortly after sulfonamides were introduced in the 1930s, suggesting that resistance could naturally occur even before the widespread use of antibiotics. However, these early instances did not yet involve deadly resistant pathogens. The widespread adoption of antibiotics marked the beginning of the antibiotic era, but human activities, including the extensive use of high antibiotic concentrations, significantly altered their efficacy and accelerated the spread of antibiotic-resistant bacteria [9].

Infections caused by both Gram-positive and Gram-negative bacteria have become increasingly challenging to treat due to multidrug resistance, which makes them unresponsive to traditional antibiotics. Antibiotic resistance has severely compromised the effectiveness of antibiotics in clinical settings throughout both the pre-antibiotic and antibiotic eras [10].

The potential for resistance to develop against any therapeutic agent limits its effectiveness [11,12]. Consequently, developing the next generation of antibiotics is crucial, as resistance undermines their therapeutic efficacy. When a pathogen shows increased resistance to a previously effective standard therapy, it is described as developing tolerance to the antibacterial agent, in this case an antibiotic [13-15].

AMR is a global crisis exacerbated by the overuse and misuse of antibiotics. The World Health Organization (WHO) has identified AMR as one of the top 10 global public health threats [16]. Bacteria resistant to multiple antibiotics are causing infections that are increasingly difficult to treat, leading to prolonged hospital stays, higher medical costs, and increased mortality. Factors contributing to AMR include inappropriate prescribing practices, the lack of adherence to treatment guidelines, and insufficient infection control measures.

The health regulator in Abu Dhabi is actively collaborating and supporting efforts to combat AMR, emphasizing the enhancement of the Emirate's resilience. This commitment is demonstrated through initiatives that prioritize emerging infectious diseases and AMR as critical areas of public health research and prevention [17]. Hence, the purpose of this study aligns with Abu Dhabi's proactive stance against AMR, which is underscored by the health regulator's efforts to prioritize research and prevention in emerging infectious diseases and AMR. By focusing on these priorities, the study aims to examine the dynamics of antibiotic stewardship and clinicians' prescribing habits pre-pandemic among non-surgical hospitalized patients and therefore contributing to the broader goal of enhancing public health preparedness and resilience in Abu Dhabi, UAE.

## Materials and methods

This retrospective observational study was conducted at the general hospital of the Abu Dhabi Emirate, spanning 12 months from March 2023 to February 2024 for data collection. Ethical approvals were obtained from the Institutional Review Board of Burjeel Holdings (BH/REC/039/22) and the Department of Health Abu Dhabi Health Research and Technology Ethics Committee (DOH/CVDC/2023/512). 

Study population

All inpatients aged 25-40 years who were not undergoing surgery between January 2019 and December 2019 were included in the study.

Eligibility criteria

This study included patients' diseases and medical conditions, as diagnosed and indicated by clinicians in patient electronic medical records (EMRs), identified through the International Classification of Diseases, 10th Revision, Clinical Modification (ICD-10-CM) codes [18] pertinent to specific diseases and medical conditions relevant to the respiratory tract, genitourinary system, nosocomial infections, and related diseases.

Surgical cases, patients under 25 and over 40 years of age, and those with other diagnoses were excluded from the study criteria.

Data collection

The following steps were taken to ensure thorough, consistent, and transparent data collection, thereby enhancing the reliability and validity of the research findings.

Identification

A comprehensive search of EMRs was conducted using predefined search terms and diagnosis codes relevant to the study. Discharge reports (Excel format) from January to December 2019 were retrieved from the hospital information system (HIS) for case identification.

Screening

Initially, identified records were reviewed to exclude duplicates and non-eligible cases based on inclusion and exclusion criteria by applying Excel (Microsoft Corp., Redmond, WA) functions. Only records with relevant infections proceeded to the next stage.

This approach was taken irrespective of whether the diagnoses were primary or secondary to eliminate several types of biases: selection bias (by including both primary and secondary diagnoses), referral bias (by capturing cases that might not be the primary reason for admission but are still relevant), observer bias (by ensuring an unbiased identification of cases, minimizing researchers' subjective judgments), confirmation bias (by not prioritizing primary over secondary diagnoses, preventing selective focus on expected cases), and exclusion bias (by avoiding the unintentional exclusion of patients with significant secondary conditions related to the study).

Eligibility

The remaining records underwent a detailed review and segregation via applying Excel functions to confirm eligibility against predefined age parameters. Inconsistencies or missing data were addressed through further examination or consultation with healthcare providers as needed.

Data Extraction

A standardized data collection Excel template was used to systematically collect patient demographics, the length of stay (LoS), diagnosis details, antibiotic administration, prescriptions, resistance/susceptibility patterns, adverse events, mortality, and clinical outcomes. Data were anonymized using unique study numbers to protect patient confidentiality.

Data Cleaning

Extracted data underwent thorough review for errors or inconsistencies. Discrepancies were corrected, and missing values were addressed to ensure a clean dataset ready for analysis, maintaining high standards of data quality and integrity.

**Figure 1:**
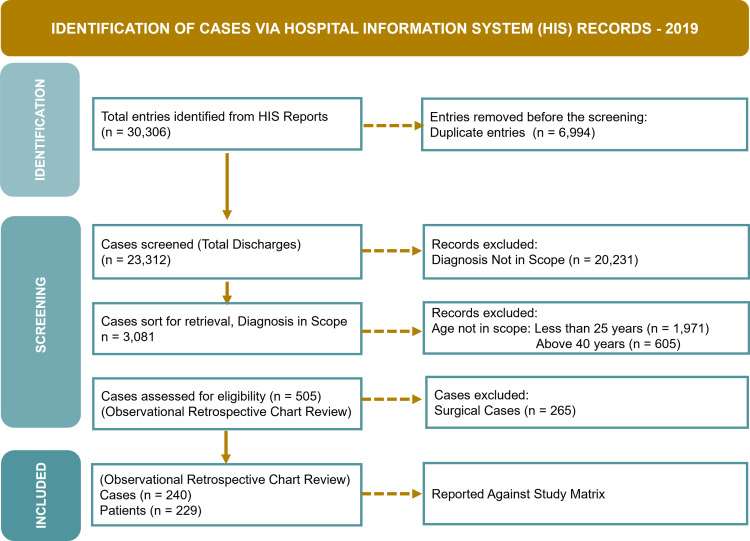
Case Selection Process for the Study (Source: Author's Own Property)

Figure 1 visually represents the steps taken to ensure a comprehensive, unbiased, and accurate selection of cases for the study.

Sample size

A total of 240 cases, representing 229 patients, were included in the study based on the eligibility criteria. The sample comprised 70 males and 170 females, with mean ages of 32.37 and 31.91 years, respectively, spanning 37 nationalities.

Key indicators for assessing study outcomes

The study examines antibiotic prescribing patterns, the maturity levels of ASP in clinical practice, and the overall burden on the health system. Referring to local and international guidelines [19-23] for establishing an ASP, Tables 1-3 outline the critical metrics and evaluation techniques for each objective: objective I, clinicians' prescribing practices and antibiotic usage; objective II, antibiotic stewardship program interventions; and objective III, impact on the healthcare system.

**Table 1:** Key Metrics and Evaluation Techniques for Objective I (Source: Author's Own Property) LoT, length of treatment; MAR, medication administration records; DoT, days of therapy

Key Indicators	Evaluation Techniques
Patient-wise antibiotic utilization analysis: inpatient admissions, at discharge, and overall duration (days)	All 240 cases were reviewed to document the total number of antibiotic days during hospitalization and discharge and overall. The duration of antibiotic use at discharge was noted from the discharge prescription.
Patient-wise antibiotic exposure and LoT (days)	All 240 cases were evaluated to document the LoT in calendar days for antibiotic therapy, with values averaged to present patient-specific data.
Antibiotic agent selection	All cases were reviewed using MAR to identify simultaneous prescriptions of multiple antibiotics for anaerobic therapy (n = 240 cases).
Antibiotic use in suspected and confirmed viral and fungal infections	Cases were reviewed using MAR and laboratory results to detect suspected or confirmed viral and fungal infections, reporting findings irrespective of antibiotic administration during inpatient stays, at discharge, or both (n = 240 cases).
Utilization of unreported antibiotics	All cases were assessed to evaluate the utilization of evidence-based antibiotics by comparing data from microbiological culture reports (susceptibility/resistance) with MAR and antibiograms (n = 240 cases).
Decision of antibiotic administration during hospitalization	Antibiotic administration data collected from the MAR for all cases were analyzed to identify instances where no antibiotics were prescribed during the patient's stay, indicating conservative management (n = 240 cases).
Antibiotic selection (empirical versus directed)	Eligible cases were assessed to determine whether the antibiotic selection at the time of admission was empirical or directed. This analysis excludes cases where interventions were made during hospitalization and after the release of microbiological culture reports (n = 220 cases, excluding 20 cases where no antibiotics were administered during hospitalization).
Accuracy of empirical decisions	Inaccuracy status was assessed by comparing microbiological culture reports with MAR, focusing on discrepancies such as antibiotic use without culture growth, non-compliance with organizational antibiogram for resistant antibiotics, or missing susceptibility information on culture reports (n = 189 cases, excluding three cases of directed therapy and 28 cases with no cultures during the stay).
Antibiotic DoT	All 240 cases underwent a comprehensive manual review to capture antibiotic administration data from the MAR. The calculation involved determining the rate of antibiotic DoT per 1,000 patient days using the following formula: antibiotic DoT rate = (total quarterly antibiotic DoT / total quarterly patient days) × 1,000. Here, "antibiotic DOT" represents the total sum of antibiotic days for all patients/cases within the facility during each quarter, and "patient days" refers to the total number of days that the patient remained hospitalized, including those within the study's scope.

**Table 2:** Key Metrics and Evaluation Techniques for Objective II (Source: Author's Own Property) MAR, medication administration records; EMR, electronic medical record

Key Indicators	Evaluation Techniques
Antibiotic therapy review for inpatients for >3 days: escalations, de-escalations, and transitions	The eligible cases were additionally scrutinized to grasp the practice trends regarding antibiotic therapy assessment during hospital stays. Since microbiological cultures are typically available within 48-72 hours, this is an opportune time to evaluate changes in therapy, including incorporating escalations/de-escalations, transitioning from intravenous (IV) to oral administration, and discontinuing antibiotics (n = 85 cases, excluding one case with no antibiotic use).
Antibiotic therapy review at discharge for >3 days: escalations, de-escalations, and transitions	The eligible cases were further examined to understand the trends in antibiotic therapy assessment at discharge. Given that microbiological culture results are typically available within 48-72 hours and available at discharge, this stage was used to evaluate therapy modifications, including therapy escalation or de-escalation, transitioning from intravenous to oral administration, and the discontinuation of antibiotics at discharge (n = 85 cases, excluding one case with no antibiotic use).
The decision of antibiotic prescribing at discharge	Data from MAR for all cases were analyzed to identify instances where no antibiotics were prescribed at discharge (n = 240 cases).
Selection of IV antibiotic at discharge	Cases, excluding those without any antibiotic treatment, were closely examined to assess the prescription of IV antibiotics at discharge. Data were collected from discharge prescriptions associated with the relevant case numbers in the patient's EMR (n = 198 cases, excluding 42 cases with no antibiotic at discharge).

**Table 3:** Key Metrics and Evaluation Techniques for Objective III (Source: Author's Own Property) AMR: antimicrobial resistance

Key Indicators	Evaluation Techniques
Average length of stay (LoS) (days)	All 240 cases were documented with their respective admission and discharge dates, allowing for the calculation of the length of stay for each patient. Subsequently, an analysis was conducted to determine the average LoS per patient.
Impact on hospital stay with empirical therapy (accurate versus inaccurate decisions)	Eligible cases were further examined to evaluate their impact on the length of stay. The statistical calculations included combining the total number of completely inaccurate and mixed decisions to reflect the overall number of inaccurate decisions (n = 189 cases, excluding three cases of directed therapy and 28 cases with no cultures during the stay).
Evaluation of side effects of antibiotic therapy	All cases were evaluated to determine the usage of probiotics or antidiarrheal drugs during hospitalization, aiming to identify those potentially experiencing side effects from prolonged antibiotic therapy (n = 220 cases, excluding 20 cases where no antibiotics were administered during hospitalization).
Identifying suspected healthcare-associated infections (HAI)/nosocomial infections	Positive cultures with various isolates were analyzed to detect HAIs, defined as those emerging at least 48 hours after admission or within 30 days post discharge. The study involved 86 cases with inpatient stays exceeding three days. Data were collected from microbiological culture reports (pus swab, body fluid, and sputum within 72-96 hours; blood after seven days; and urine within 48-72 hours) and organized by the reported date (n = 86 cases with >3 days of inpatient stay).
Identifying 30-day readmissions following initial treatment and assessing their impact	The study assessed 229 patients for readmissions within 30 days, focusing solely on financial impacts related to health insurance settlements, excluding staffing or other hospital costs.
Antibiotics susceptibility/resistance rates	The institutional antibiograms were utilized to track AMR and susceptibility trends throughout the study to uncover correlations between local antibiotic prescribing practices and infection control measures by comparing profiles across various periods.

Statistical analysis

Statistical analysis was performed using several Excel functions such as sum, division, average, Countif, multiplication, and percentage. Additionally, WHONET software (Brigham and Women's Hospital, Boston, MA) was utilized for calculating the percentage of susceptibility.

The length of treatment (LoT) (days) was calculated to determine the number of days each patient received antibiotics during hospital stay, irrespective of the number of different drugs. The LoT for each patient was then averaged to provide an overall average LoT per patient.

The calculation of antibiotic days (i.e., the total number of days a patient receives antibiotics) involved summing the duration of each antibiotic administered to a patient and then aggregating these durations for all antibiotics received during hospitalization. The total antibiotic days for all patients were then compiled using the Excel functions to produce quarterly data. The antibiotic days of therapy (DoT) rate was subsequently calculated using the formula provided in Table 1.

The readmission analysis focused on readmissions occurring within 30 days of the initial care. This was determined by calculating the number of days between the discharge date and the readmission date, using Excel functions for accuracy. Each eligible case was counted based on unique medical record numbers, ensuring that multiple case numbers with the same medical record number were considered as a single patient.

The antibiogram was analyzed using the WHONET software, and non-duplicate isolates were chosen.

The statistical analysis of other key metrics was conducted using the evaluation methods outlined in Tables 1-3. Microsoft Excel and its functions served as the primary tools for these calculations. The analysis involved using the number of eligible cases that met the criteria for each indicator as the numerator, with the total number of cases as the denominator (denoted as "n" against each indicator) in Tables 1-3. This ratio was then multiplied by 100 to produce a percentage figure, offering a statistical representation of the findings.

## Results

Clinicians' prescribing practices and antibiotic usage

The average duration of antibiotic use per patient during inpatient admissions is 5.32 days, while at discharge, it is 7.14 days. Overall, including both inpatient admissions and discharge, the average duration of antibiotic therapy per patient is 6.23 days.

All 240 cases were assessed for antibiotic exposure, i.e., LoT, resulting in an average of 2.87 days per patient.

In 11 cases (4.58%, n = 240), duplicate anaerobic therapy was identified, involving simultaneous prescriptions of various antibiotic combinations: meropenem with metronidazole; metronidazole with ceftolozane sulfate and tazobactam; vancomycin, moxifloxacin, and meropenem; and metronidazole with piperacillin and tazobactam.

Using medication administration records (MAR) and laboratory results, all cases (n = 240) were reviewed to detect suspected or confirmed viral/fungal infections. Results showed 41 cases (17.08%), reported regardless of antibiotic administration during inpatient stays, discharge prescriptions, or both.

The further evaluation of these 240 cases to assess the utilization of evidence-based antibiotics involved comparing data from microbiological culture reports (susceptibility/resistance) with MAR and antibiograms. The analysis revealed that antibiotics such as macrolides (clarithromycin), first-generation cephalosporins (cephalexin), colistimethate, colistin, and metronidazole were administered. However, these antibiotics were neither documented in the culture reports nor included in the facility's antibiogram.

Regarding the decision of antibiotic administration during hospitalization (n = 240), the findings indicate that 20 cases (8.33%) did not receive antibiotics throughout their hospital stay. In contrast, 220 cases (91.66%) received antibiotics within 24 hours of admission. Additionally, 13 cases (5.41%) did not receive antibiotics during their hospital stay and were discharged without antibiotic prescriptions.

All cases (n = 220, excluding 20 cases with no antibiotic use during the stay) were assessed for initial antibiotic selection at admission. Results indicate that 217 cases (98.63%) received empirical therapy, while three cases (1.36%) received directed therapy at admission, excluding interventions made post hospitalization and after microbiological culture reports.

The review of empirical decision accuracy (n = 189, excluding 28 cases with no cultures) resulted in 141 cases (74.60%) with inaccurate decisions across 286 episodes of antibiotic selection. Accurate decisions were observed in 25 cases (13.2%), while 23 cases (12.16%) had mixed episodes, including 35 instances of inaccurate prescribing/administration and 25 instances of accurate episodes. An "episode" refers to each occurrence of antibiotic administration per patient, irrespective of specific details such as duration, dose, or frequency.

**Figure 2:**
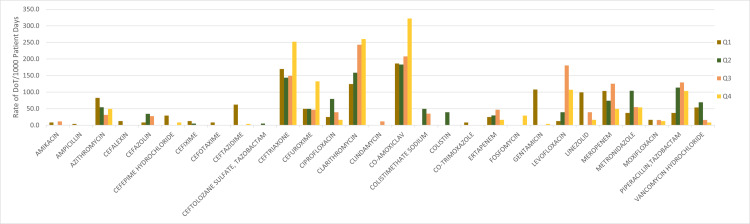
DoT/1,000 Patient Days: Correlation With Prescribing Habits (Source: Author's Own Property) Q1, quarter 1 (January-March) 2019; Q2, quarter 2 (April-June) 2019; Q3, quarter 3 (July-September) 2019; and Q4, quarter 4 (October-December) 2019 DoT: days of therapy

The DoT rate per 1,000 patient days (Figure 2) correlates with prescribing habits.

Antibiotic stewardship program interventions

Out of 85 cases reviewed for antibiotic use in patients with stays exceeding three days, findings revealed varying levels of stewardship conduct. Only two cases (2.35%) demonstrated "appropriate stewardship conduct." In contrast, 48 cases (56.47%) were categorized as "inappropriate stewardship conduct," characterized by inadequate responses to microbial culture results, such as using multiple sensitive agents and lacking de-escalation or transition from intravenous (IV) to oral antibiotics. Inappropriate practices included the use of antibiotics not recommended by culture reports, the continuation of resistant agents, and failure to discontinue or adjust therapy despite negative culture results. Additionally, 35 cases (41.17%) were classified as "nonevidence-based practice," with culture reports either released after discharge or not conducted at all.

Upon reviewing these cases (n = 85) at discharge, the findings revealed varying levels of stewardship conduct. Among those reviewed, 14 cases (16.47%) exhibited "appropriate stewardship conduct." In contrast, 44 cases (51.76%) were categorized as "inappropriate stewardship conduct," marked by inadequate responses to microbial culture results. This included instances where patients were discharged with antibiotics despite negative culture reports, prescribed antibiotics not recommended by culture results, continued on resistant antibiotics, or given multiple sensitive antibiotics. Additionally, 27 cases (31.76%) were classified as "nonevidence-based practice," with culture reports released after discharge or no culture conducted at all in some cases.

The review of antibiotic prescribing at discharge, involving 240 cases, showed diverse patterns: among these cases, 42 (17.5%) had no antibiotics prescribed upon discharge. The majority, comprising 198 cases (82.5%), received prescriptions for antibiotics at discharge, typically ranging from one to three antibiotics per case.

Out of 198 cases (82.5%) where antibiotics were prescribed at discharge, five cases (2.52%) received IV antibiotics. This excludes the 42 cases where no antibiotics were prescribed at discharge.

Impact on the healthcare system

Recording specific admission and discharge dates for each case enabled the calculation of the LoS per patient, revealing an average of 3.86 days.

Out of 189 cases identified for empirical therapy with cultures, further scrutiny revealed that accurate empirical decisions were associated with an average LoS of 3.36 inpatient days, whereas inaccurate empirical decisions had an average length of stay of 4.45 inpatient days.

Excluding the 20 patients who did not receive antibiotics during their stay, three cases (1.36%, n = 220) received probiotics or antidiarrheal agents during their hospitalization and antibiotic treatment.

Four patients (1.74% of the total 229) were readmitted within 30 days of their initial care, resulting in an additional cost of approximately $18,927.51. This analysis focuses exclusively on financial settlements with patients' health insurance companies, excluding considerations of staffing or other direct and indirect hospital costs.

Among them, seven cases are suspected of having healthcare-associated infections (HAI)/nosocomial infections, involving pathogens such as *Escherichia coli*,* Pseudomonas *species,* Bacillus *species,* Klebsiella pneumoniae*, coagulase-negative* Staphylococcus *species, and* Enterococcus *species.

**Figure 3:**
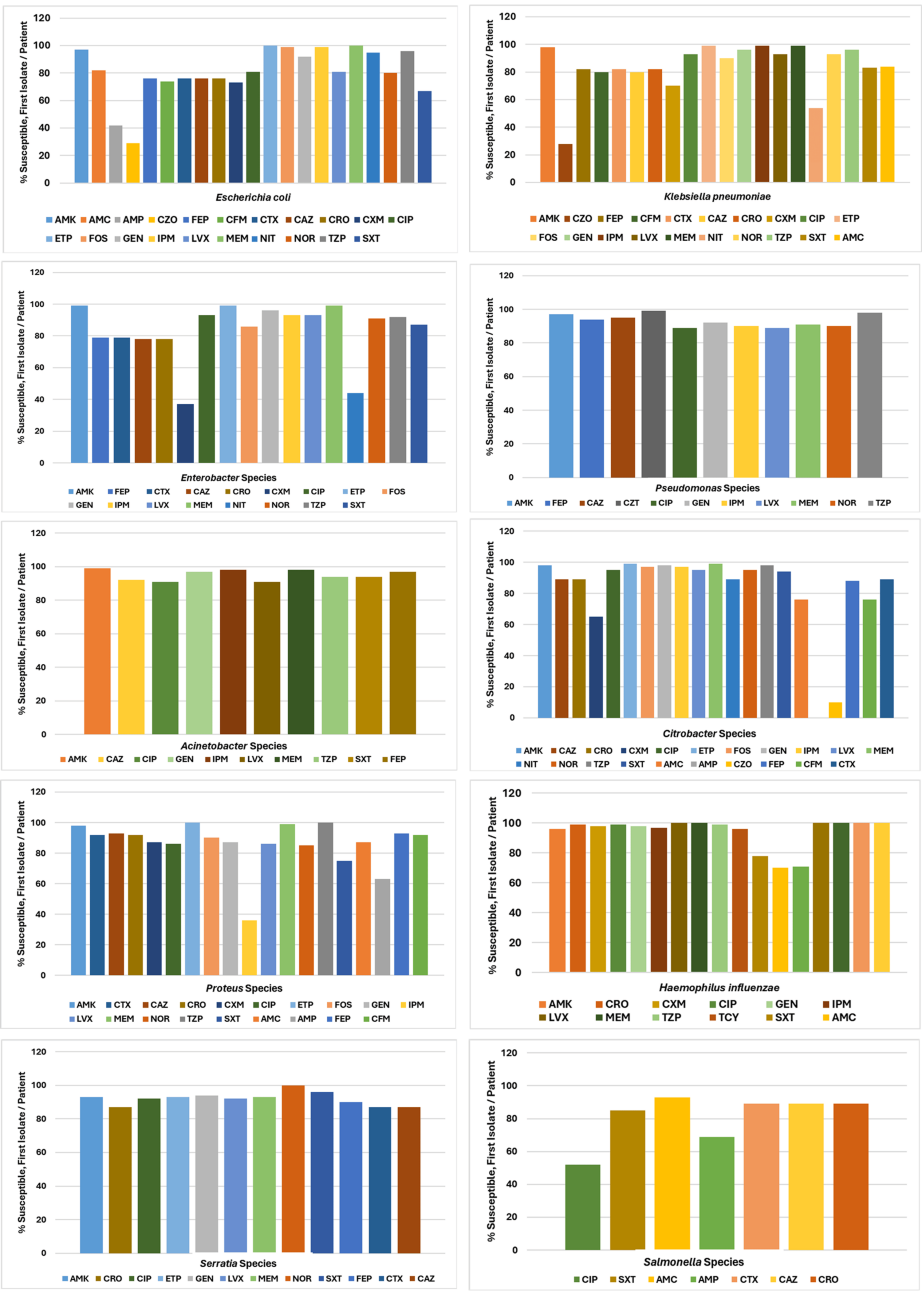
Antibiogram Gram-Negative Organisms, 2019 (Source: Data From Institutional Antibiogram; Graphical Representation; Author's Own Property) AMK, amikacin; CZT, ceftolozane/tazobactam; LVX, levofloxacin; AMC, amoxicillin/clavulanic acid; CRO, ceftriaxone; MEM, meropenem; AMP, ampicillin; CXM, cefuroxime; NIT, nitrofurantoin; CZO, cefazolin; CIP, ciprofloxacin; NOR, norfloxacin; FEP, cefepime; ETP, ertapenem; TZP, piperacillin/tazobactam; CFM, cefixime; FOS, fosfomycin; TCY, tetracycline; CTX, cefotaxime; GEN, gentamicin; SXT, trimethoprim/sulfamethoxazole; CAZ, ceftazidime; IPM, imipenem

**Figure 4:**
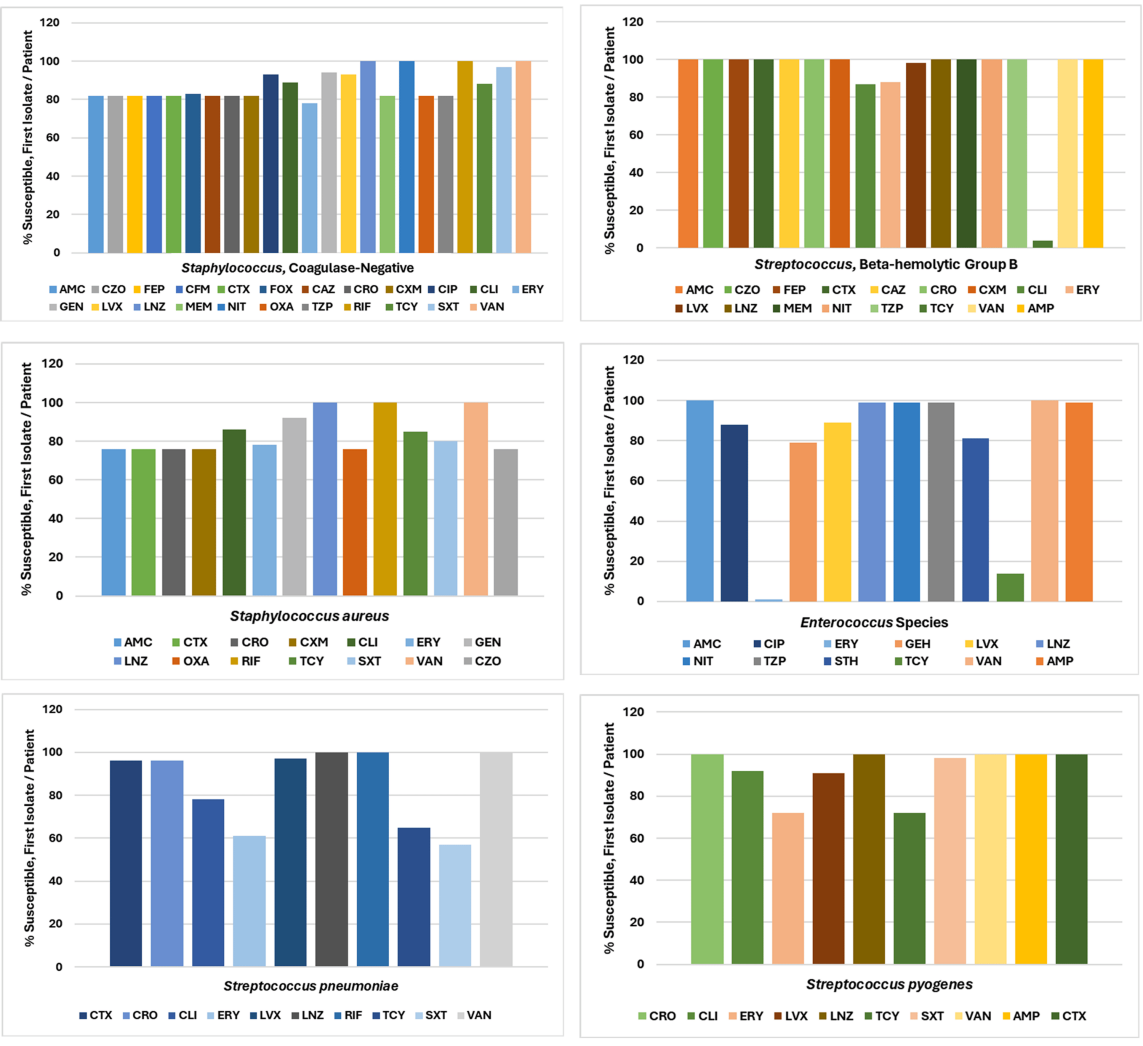
Antibiogram Gram-Positive Organisms, 2019 (Source: Data From Institutional Antibiogram; Graphical Representation; Author's Own Property) AMC, amoxicillin/clavulanic acid; CXM, cefuroxime; NIT, nitrofurantoin; AMP, ampicillin; CIP, ciprofloxacin; OXA, oxacillin; CZO, cefazolin; CLI, clindamycin; TZP, piperacillin/tazobactam; FEP, cefepime; ERY, erythromycin; RIF, rifampin; CFM, cefixime; GEH, gentamicin-high; STH, streptomycin-high; CTX, cefotaxime; GEN, gentamicin; TCY, tetracycline; FOX, cefoxitin; LVX, levofloxacin; SXT, trimethoprim/sulfamethoxazole; CAZ, ceftazidime; LNZ, linezolid; VAN, vancomycin; CRO, ceftriaxone; MEM, meropenem

Figure 3 depicts the antibiogram for Gram-negative organisms in 2019, while Figure 4 illustrates the antibiogram for Gram-positive organisms in 2019, indicating the percent of susceptible and first isolate/patient.

*Escherichia ​​​​​*​​*coli *showed reduced susceptibility (≤80%) to ampicillin (AMP), cefazolin (CZO), cefepime (FEP), cefixime (CFM), cefotaxime (CTX), ceftazidime (CAZ), ceftriaxone (CRO), cefuroxime (CXM), and trimethoprim/sulfamethoxazole (SXT). *Klebsiella pneumoniae* also displayed decreased susceptibility (≤80%) to CZO, CFM, CAZ, CXM, and nitrofurantoin (NIT). *Enterobacter *specieshad reduced susceptibility (≤80%) to FEP, CTX, CAZ, CRO, CXM, and NIT. *Proteus *species showed decreased susceptibility (≤80%) to AMP, imipenem (IPM), and SXT. *Citrobacter *species exhibited 100% resistance to AMP and only 10% susceptibility to CZO. *Haemophilus influenzae* was mostly susceptible to antibiotics but had declining susceptibility (≤80%) to amoxicillin/clavulanic acid (AMC), AMP, and STX. *Salmonella *species were generally susceptible to most antibiotics, though their susceptibility to AMP and ciprofloxacin (CIP) was declining (≤80%).

*Staphylococcus *(coagulase-negative) was susceptible to most antibiotics. *Streptococcus *(beta-hemolytic group B) also showed susceptibility to most antibiotics but had less than 10% susceptibility to tetracycline (TCY). *Staphylococcus aureus* displayed declining susceptibility (less than 80%) to various antibiotics including AMP, CZO, CTX, CRO, CXM, erythromycin (ERY), and oxacillin (OXA). *Enterococcus *species exhibited less than 20% susceptibility to TCY and ERY. *Streptococcus pneumoniae* showed declining susceptibility (less than 80%) to clindamycin (CLI), ERY, TCY, and SXT. *Streptococcus pyogenes *was generally susceptible to most antibiotics but had declining susceptibility (less than 80%) to ERY and TCY.

## Discussion

The health regulator of the Emirate of Abu Dhabi has issued guidelines for ASP to aid healthcare facilities in enhancing antimicrobial prescribing practices, particularly for antibiotics, and to curb the development and spread of resistant bacterial strains within healthcare facilities and communities [19]. Given the rising need for robust reporting, the health regulator has also recently established a standard for the monitoring and reporting of AMR. This standard sets the requirements for a laboratory-based surveillance system for AMR in Abu Dhabi and outlines the obligations for healthcare facilities to collect, monitor, and report antimicrobial susceptibility testing (AST) results and related data [20]. Our findings further underscore the necessity for a comprehensive monitoring and reporting system to effectively combat AMR.

A recent report on AMR trends in Abu Dhabi from 2010 to 2022 indicates that AMR is high and/or increasing in the Emirate, particularly for methicillin-resistant *Staphylococcus aureus *(MRSA),extended-spectrum beta-lactamase (ESBL)* E. coli*,* and *ESBL* K. pneumoniae*, which is notably higher compared to western European countries. The report also identified critical priority pathogens for the Abu Dhabi region, including *Acinetobacter baumannii*,* Pseudomonas aeruginosa*,allEnterobacterales,* E. coli*, and *Klebsiella pneumoniae* [21]. These findings align with our study results, emphasizing the urgent need for comprehensive strategies to effectively address AMR.

The study found that the antibiotics were used in duplicate in 5% of cases, despite recommendations to review antibiotic therapy to avoid unnecessary duplication, particularly the use of agents with overlapping spectra. The combination of two agents with anaerobic activity is generally unnecessary [22,23]. Consequently, these prescribing practices should be reviewed.

Optimizing antibiotic use is essential for effectively treating infections, protecting patients from the harms of unnecessary antibiotic use, and combating antibiotic resistance. ASPs help clinicians improve clinical outcomes and minimize harm by enhancing antibiotic prescribing practices [24,25]. Hospital antibiotic stewardship programs can increase infection cure rates while reducing treatment failures, *Clostridioides difficile* infections, adverse effects, antibiotic resistance, hospital costs, and lengths of stay [25-27].

Upon reviewing the inaccuracies in empirical therapy decisions, which were assessed by comparing microbiological culture reports with MAR, significant discrepancies were identified. These discrepancies included the continuation of antibiotic use despite no culture growth, non-compliance with the organizational antibiogram for resistant antibiotics, and the use of antibiotics even when susceptibility information was missing from culture reports. The analysis revealed that 74.60% of decisions were inaccurate, which is substantially higher than the approximately 30% of all antibiotics prescribed in US acute care hospitals that are deemed either unnecessary or suboptimal [24,28].

Our study findings emphasize the need for additional efforts to improve clinicians' prescribing habits and adherence to stewardship conduct. These efforts are crucial for advancing the maturity level of existing ASPs, supporting the fight against AMR, and reducing hospital stays, readmissions, and adverse effects.

Limitations

The study's retrospective design limits causal inference and may introduce biases typical of retrospective analyses. Reliance on EMRs may lead to missing data, coding errors, and variations in record-keeping, affecting data accuracy. Generalizability is restricted to specific healthcare settings and patient demographics. The study's focus on hospitalized patients and specific infections may exclude outpatient data and broader infection types. Additionally, limitations in generating reports on staff involvement in patient care restricted financial impact analysis to claims submitted to health insurance. The results rely entirely on the data accessible in the EMR, including MAR and culture reports. The study does not question clinical decisions as it is confined to ideal stewardship practices and related interventions.

Overall, this study highlights key aspects of antibiotic prescribing practices and resistance patterns among hospitalized patients in Abu Dhabi, UAE. The findings underscore the need for enhanced antimicrobial stewardship efforts to enhance treatment efficacy, mitigate resistance emergence, and improve patient outcomes. By implementing targeted interventions and fostering a culture of evidence-based prescribing, healthcare systems can better combat the growing threat of AMR and ensure sustainable healthcare delivery.

Further research is advised to explore how these findings enhance our comprehension of the effects of the COVID-19 pandemic on antibiotic stewardship practices, resistance patterns, and clinician prescribing behaviors.

## Conclusions

The study reveals alarming trends in antibiotic resistance and prescribing practices that demand urgent attention. High rates of inappropriate antibiotic usage, suboptimal administration patterns, and frequent inaccurate decision-making underscore the growing challenge of AMR. These findings emphasize the critical need of strengthening the ASP efforts. By promoting adherence to ASP guidelines, enhancing education on prudent antibiotic use, and implementing effective monitoring and intervention strategies, healthcare providers can mitigate the spread of resistance, improve patient care outcomes, and safeguard public health. Addressing these issues proactively is essential in combating the escalating threat of AMR and ensuring sustainable antibiotic efficacy for future generations.
